# Breastfeeding and timing of pubertal onset in girls: a multiethnic population-based prospective cohort study

**DOI:** 10.1186/s12887-019-1661-x

**Published:** 2019-08-09

**Authors:** Sara Aghaee, Julianna Deardorff, Louise C. Greenspan, Charles P. Quesenberry, Lawrence H. Kushi, Ai Kubo

**Affiliations:** 1 Kaiser Permanente Division of Research, 2000 Broadway, Oakland, CA 94612 USA; 2 Division of Maternal and Child Health, University of California, Berkeley, School of Public Health, 2121 Berkeley Way #5302, Berkeley, CA 94720 USA; 3 Kaiser Permanente San Francisco Medical Center, 2425 Geary Boulevard, San Francisco, CA 94115 USA

**Keywords:** Developmental origin of health and diseases, Breastfeeding, Obesity, Puberty

## Abstract

**Background:**

Early puberty is associated with higher risk of adverse health and behavioral outcomes throughout adolescence and adulthood. US girls are experiencing earlier puberty with substantial racial/ethnic differences. We examined the association between breastfeeding and pubertal timing to identify modifiable risk factors of early puberty and potential sources of racial/ethnic differences in the timing of pubertal development.

**Methods:**

A prospective cohort study of 3331 racially/ethnically diverse girls born at Kaiser Permanente Northern California (KPNC) between 2004 and 06. All data were obtained from KPNC electronic clinical and administrative datasets. Mother-reported duration of breastfeeding was obtained from questionnaires administered at each ‘well-baby’ check-up exam throughout the baby’s first year and categorized as ‘Not breastfed’, ‘Breastfed < 6 months’, and ‘Breastfed ≥ 6 months’. Pubertal development data used Tanner stages assessed by pediatricians during routine pediatric checkups starting at age 6. Pubertal onset was defined as transition from Tanner Stage 1 to Tanner Stage 2+ for breast (thelarche) and pubic hair (pubarche). Weibull regression models accommodating for left, right, and interval censoring were used in all analyses. Models were adjusted for maternal age, education, race/ethnicity, parity and prepubertal body mass index (BMI). We also examined race/ethnicity as a potential effect modifier of these associations.

**Results:**

Not breastfeeding was associated with earlier onset of breast and pubic hair development compared to breastfeeding ≥6 months (adjusted hazard ratio [HR]: 1.25; 95% confidence interval [CI]: 1.07–1.46; HR: 1.24; 95% CI: 1.05–1.46, respectively). Breastfeeding for < 6 months was also associated with the risk of earlier pubic hair development (HR: 1.14; 95% CI: 1.00–1.30, compared to breastfeeding ≥6 months). Inclusion of girls’ prepubertal BMI slightly attenuated the association between breastfeeding and timing of breast onset but remained significant. The association between not breastfeeding and early breast development may be stronger among African American girls (HR: 1.92; 95% CI: 1.01–3.66, no breastfeeding vs. ≥6 months) than other racial/ethnic groups.

**Conclusions:**

Breastfeeding is an independent predictor of pubertal onset in girls, and the strength of the association may vary by race/ethnicity. Providing breastfeeding support and lactation education for high risk mothers may help prevent earlier pubertal onset and promote positive health outcomes later in life.

## Background

Girls in the United States are experiencing earlier pubertal onset and sexual development than in the past [1]. Girls who undergo earlier puberty are at higher risk of emotional and behavioral problems including depression, eating disorders, substance use, early initiation of sexual behavior, teen pregnancy, and sexual abuse [2–7]. They are also at increased risk for serious health conditions later in life, including reproductive cancers and cardiovascular disease [2, 8]. Prepubertal obesity has been associated with earlier pubertal development, but does not fully explain the overall decline in the age of pubertal onset as this trend has been observed in girls of varying body mass index (BMI) [1]. We recently reported that African American girls experience substantially earlier pubertal onset than non-Hispanic White or Asian girls. We found that the median age of breast onset was 8.8 years in African American girls, compared to 9.7 years in White girls [1]. This difference in age is particularly striking given that just a century ago African American girls experienced later menarche than their non-Hispanic White counterparts [9]. Racial/ethnic differences in age of pubertal onset may lead to worse health outcomes for racial/ethnic minorities in the US; thus, understanding these differences is crucial to efforts to narrow racial/ethnic health disparities.

Infant nutrition, including breastfeeding, may offer a partial explanation for racial/ethnic disparities in age of pubertal onset. Studies show that African American mothers are the least likely to initiate breastfeeding [10, 11]. One study found that only 47.9% of African American children were ever breastfed, compared with 74% of non-Hispanic Whites and 71.5% of Hispanics, among those born to US-born parents [10]. When examining breastfeeding practices at 6 months, breastfeeding rates were similarly substantially lower in the African American group, at 21.3%, compared with non-Hispanic Whites (39%), and Hispanics (32.3%) [10]. Because of the known association between lack of breastfeeding and obesity as well as the association between childhood obesity and early puberty, [1, 12] differences in breastfeeding practices may at least partially contribute to the observed disparities in pubertal timing among different racial/ethnic groups.

Few studies have examined the association between breastfeeding and timing of puberty, and most were small and/or had racially/ethnically homogenous cohorts [13–21]. We thus conducted a population-based study using a large, racially/ethnically diverse cohort of girls and prospectively-collected, clinical data on pubertal development to investigate whether breastfeeding is associated with the timing of pubertal onset. We hypothesized that girls who were breastfed for longer durations would experience later pubertal onset compared to those who were not breastfed. We also examined the role of prepubertal BMI in the relationship between breastfeeding and timing of puberty. Lastly, we explored whether there are differences in the strengths of the associations among different race/ethnic groups.

## Methods

### Participants

The study was conducted within Kaiser Permanente Northern California (KPNC), a large integrated health care delivery system that serves a socio-demographically and ethnically diverse population of over 4.2 million in Northern California. Using the KPNC clinical and administrative databases, including its electronic health record (EHR) system, we identified a cohort of mother-daughter pairs who met the following eligibility criteria. All girls were born at a KPNC medical facility between 2004 and 2006 and were followed through March 31, 2018. Girls were included if they had continuous KPNC membership, defined as KPNC health insurance with no coverage gap > 90 days during the follow-up period, to minimize missing data. We included mother-daughter pairs where the mother did not have extreme BMI (< 15 kg/m^2^ or > 60 kg/m^2^) and had attended at least 1 postnatal pediatrician checkup when their daughter was < 6 months and at least another checkup at ≥6 months. Additionally, girls were required to have had at least one measured prepubertal BMI and one pubertal stage (breast and/or pubic hair) assessment as described below.

Girls with medical conditions known to influence pubertal development, such as adrenal tumors, or premature birth (< 37 weeks gestation) were excluded. Additionally, underage mothers (< 18 years) were excluded as they may be at greater risk for depression [22] and preterm delivery [23], both of which may influence pubertal development. Likewise, multiple births were excluded because of their association with low birthweight and preterm delivery [24]. Lastly, we excluded pairs that were missing important covariates such as maternal age at delivery and race/ethnicity.

A total of 3331 mother-daughter pairs were included in the analysis after excluding 45 who had conditions affecting pubertal development, 382 premature births, 182 multiple births, 22 underage mothers, and 28 pairs with missing information on important covariates. All data were drawn from KPNC EHR. The study was approved by the KPNC Institutional Review Board.

### Measurements

#### Exposure variable (breastfeeding duration)

KPNC members with newborn babies are strongly encouraged to attend “Well Baby/Child” visits to discuss and assess their child’s health and growth trajectory with their pediatricians from birth to adolescence. These visits are accompanied by health questionnaires asking questions on nutrition, safety, parenting behaviors, household circumstances, and other topics pertaining to the child’s health and wellbeing. Within the first year after birth, parents are encouraged to come to these well-baby checkups at the following intervals: birth-1 week, 2–4 weeks, 2 months, 4 months, 6 months, and 9–10 months. During these checkups, parents or caregivers are asked “Do you feed your baby breastmilk?” We categorized breastfeeding practice as: “breastfed for ≥6 months” if the response was “Yes” at ≥6 months after birth; “breastfed for <6 months” if the response was “Yes” at <6 months and “No” at ≥6 months checkup; and “not breastfed” if the response was “No” to the breastfeeding questions at both <6 months and ≥ 6 months. The 6-month cutoff was selected in accordance with the World Health Organization (WHO) breastfeeding guidelines, which recommend that infants are breastfed for at least 6 months [25].

#### Outcomes

Pubertal stage for breast and pubic hair development was assessed by physical exam using the 5-level Tanner stages developed by Marshall and Tanner [26]. Beginning in 2010, documentation of Tanner stages in the KNPC EHR became a routine part of pediatric checkups. KPNC pediatricians determine girls’ breast Tanner staging using palpation and visual inspection, and pubic hair Tanner staging using visual inspection. KPNC pediatricians are trained to assess Tanner stages for all children age 6+ years during each well-child check-up exam, which are due every 1–2 years. Although KPNC encourages all pediatricians to conduct Tanner assessment and enter the data in the EHR, some pediatricians skip the assessments due to lack of time, refusal by the patients, or obvious full maturation. In our cohort the number of pediatric check-ups with a recorded Tanner Stage ranged from 1 to 7 with a median of 2 (SD: 0.9).

In this study, our primary outcome was the timing of transition from Tanner Stage 1 (prepubertal) to Tanner Stage 2 and above, which characterizes the physical and clinical onset of pubertal development. We have measured and confirmed the validity of using Tanner-staging data from the KPNC EHR, as described elsewhere [27]. Thelarche is defined as onset of breast development and pubarche is onset of pubic hair development.

#### Covariates

Girl’s prepubertal BMI was identified from clinic visits in the time window within 60 days prior to or after the last known Tanner Stage 1 assessment. Percentiles and z-scores were calculated using age- and sex-specific CDC year 2000 standard population distributions [28]. We assessed the role of prepubertal BMI in secondary analyses using continuous prepubertal BMI percentiles.

We accounted for potential confounding using several sociodemographic variables and maternal characteristics that have been associated with breastfeeding practice and pubertal development in girls [1, 10, 16, 29–32]. Maternal race/ethnicity was categorized as ‘African American’, ‘Hispanic’, ‘Asian/Pacific Islander’, and ‘non-Hispanic White’. Socioeconomic status, particularly income, is a strong predictor of pubertal outcomes, however is not collected routinely by KPNC health practitioners. Thus, we used maternal education (high school or less, some college, 4-year college, and graduate school) as a proxy for socioeconomic status. Maternal age at delivery was measured in years, and parity was categorized as ‘0’, ‘1,’, and ‘2+’. All covariates were obtained from the KPNC EHR.

### Statistical analyses

We analyzed the association between breastfeeding and age at onset of puberty using Weibull regression, an accelerated failure time and proportional hazards regression model that accommodates left, right, and interval censoring. A subject was considered left-censored if they had already transitioned to Tanner Stage 2+ at the time of the first (baseline) Tanner stage exam and right-censored if they had not transitioned to Tanner Stage 2+ by the time of the last exam or had only one assessment at Tanner Stage 1. Weibull regression analyses provided two estimates of the association of an exposure variable with the outcome of time to puberty onset: the time ratio (TR) and the hazard ratio (HR). TR estimates represent the ratio of the median time to event for a given level of the exposure variable (e.g., not breastfed) in relation to its reference level (e.g., breastfed ≥6 months). Clinically important confounders including maternal age at delivery, race/ethnicity, education, and parity were included in all models. Due to theoretical or empirical associations with breastfeeding and/or pubertal timing, birthweight was also tested as a potential covariate but was not included in the final models because its inclusion did not substantially change effect estimates.

In secondary analyses, we assessed the role of girl’s prepubertal BMI (percentile) by comparing the effect estimates for breastfeeding before and after inclusion of this variable. Effect modification by maternal race/ethnicity was also examined using a cross-product term of mother’s race/ethnicity and breastfeeding. Although the test for interaction was not statistically significant, we conducted and presented the results from the stratified analyses by race/ethnicity. All analyses were conducted using SAS version 9.4 (SAS Institute, Cary, NC).

## Results

### Participant characteristics

In this cohort of 3331 mother-daughter pairs, approximately 15% (*n* = 511) of the mothers did not breastfeed, 34% (*n* = 1123) breastfed < 6 months, and 51% (*n* = 1697) breastfed ≥6 months (Table 1). The population was racially and ethnically diverse: 24% were Asian/Pacific Islander, 26% Hispanic, and 8% African American. Compared with women who breastfed, women who did not breastfeed were more likely to be younger (*p* < 0.001), have lower levels of educational attainment (*p* < 0.001), have greater parity (*p* = 0.02), had higher BMI (*p* < 0.01) and be of Hispanic or African American race/ethnicity (*p* < 0.001). Girls who were not breastfed were more likely to be overweight compared to those who were breastfed (*p* < 0.01).

**Table 1:** Maternal and girls’ characteristics by breastfeeding duration: KPNC puberty study

Breastfeeding duration	Not breastfed	< 6 months	≥ 6 months	*P*- value
*N* = 3331 (mother-daughter pairs)	511	1123	1697	
Maternal characteristics	N (%) or Mean (SD)			
Age at delivery (years)	29.3 (5.7)	30.3 (5.4)	31.3 (5.1)	< 0.001
Maternal BMI (kg/mm^2^)				
< 25	179 (37.0)	474 (43.7)	736 (46.6)	< 0.001
25- < 30	137 (28.3)	355 (32.7)	486 (30.8)	
≥ 30	168 (34.7)	256 (23.6)	356 (22.6)	
Race/Ethnicity				
Non-Hispanic White	171 (33.5)	434 (38.7)	797 (47.0)	< 0.001
Asian/Pacific Islander	127 (24.9)	307 (27.3)	363 (21.4)	
Hispanic	149 (29.2)	294 (26.2)	439 (25.9)	
African American	64 (12.5)	88 (7.8)	98 (5.8)	
Parity				
0	204 (39.9)	504 (44.9)	686 (40.4)	0.02
1	179 (35.0)	405 (36.1)	654 (38.5)	
2+	128 (25.1)	214 (19.1)	357 (21.0)	
Education				
High school or less	190 (37.2)	342 (30.5)	400 (23.6)	< 0.001
Some college	187 (36.6)	375 (33.4)	478 (28.2)	
College/university	93 (18.2)	281 (25.0)	513 (30.2)	
Postgraduate	41 (8.0)	125 (11.1)	306 (18.0)	
Girls’ characteristics	N (%) or Mean (SD)			
Pre-pubertal BMI				
> 85th percentile	180 (35.2)	355 (31.6)	469 (27.6)	< 0.01

*BMI* Body mass index, *SD* Standard deviation

### Primary analyses

#### Breastfeeding and thelarche

After adjusting for maternal age at delivery, race/ethnicity, education, and parity, breastfeeding was associated with later age at thelarche (*p* = 0.014). Girls who were not breastfed were more likely to experience earlier thelarche compared with girls who were breastfed ≥6 months (HR: 1.25; 95% CI: 1.07–1.46) (Table 2). The time ratio estimate corresponds to approximately 2.5 months earlier onset comparing these groups. Girls breastfed < 6 months were at a slightly higher risk of earlier thelarche, however results were not statistically significant (HR: 1.09 CI: 0.97–1.22).

**Table 2:** Associations between breastfeeding duration and timing of breast development in daughters: KPNC puberty study (*N*=3,270)

Breastfeeding duration^a^	n	TR	95% CI	HR	95% CI
Not breastfed	499	0.98	0.96–0.99	1.25	1.07–1.46
< 6 months	1102	0.99	0.98–1.00	1.09	0.97–1.22
≥ 6 months	1669	Ref		Ref	

*CI* Confidence interval, *HR* Hazard ratio, *KPNC* Kaiser Permanente Northern California, *TR* Time ratio

^a^Adjusted for maternal race/ethnicity, maternal age at delivery, education, and parity

#### Breastfeeding and pubarche

There was a similar relationship between duration of breastfeeding and timing of pubarche (*p* = 0.021). Girls who were not breastfed were at 24% higher risk of earlier pubarche than girls who were breastfed ≥6 months, while girls who were breastfed for < 6 months were at 14% greater risk for earlier pubarche than those who breastfed for ≥6 months (HR: 1.24; 95% CI: 1.05–1.46, HR: 1.14; 95% CI: 1.00–1.30, respectively) (Table 3). The estimated time ratios correspond to an approximately 2.5 months earlier pubarche for daughters who were not breastfed and approximately 1.5 month earlier for daughters who breastfed < 6 months, compared with daughters who breastfed ≥6 months.

**Table 3:** Associations between breastfeeding duration and timing of pubic hair development in daughters: KPNC puberty study (*N*=3,087)

Breastfeeding duration^a^	N	TR	95% CI	HR	95% CI
Not breastfed	470	0.98	0.96–1.00	1.24	1.05–1.46
< 6 months	1028	0.99	0.97–1.00	1.14	1.00–1.30
≥ 6 months	1589	Ref		Ref	

*CI* Confidence interval, *HR* Hazard ratio, *KPNC* Kaiser Permanente Northern California, *TR* Time ratio

^a^Adjusted for maternal race/ethnicity, maternal age at delivery, education, and parity

### Secondary analyses

#### Role of prepubertal BMI

Including girl’s prepubertal BMI slightly attenuated the associations between breastfeeding duration and age at pubertal onset (thelarche: *p* = 0.099; pubarche: *p* = 0.152) (Table 4). However, the association between no breastfeeding and early thelarche remained statistically significant (HR: 1.18; 95% CI: 1.01–1.38).

**Table 4:** Associations between breastfeeding duration and pubertal development, adjusted for prepubertal BMI: KPNC puberty study

Breastfeeding duration	N	Breast^a^	Pubic Hair^a^			
		HR	95% CI	N	HR	95% CI
Not breastfed	497	1.18	1.01–1.38	470	1.13	0.96–1.34
< 6 months	1096	1.07	0.95–1.21	1023	1.12	0.98–1.28
≥ 6 months	1667	Ref		1587		Ref

*CI* Confidence interval, *HR* Hazard ratio, *KPNC* Kaiser Permanente Northern California

^a^Also adjusted for maternal race/ethnicity, maternal age at delivery, education, and parity

#### Effect modification by race/ethnicity

Although the test for interaction using cross-products was not statistically significant, we conducted stratified analyses by race/ethnicity to explore whether there may be differences in the strengths of the associations (Table 5). Although many of the examined results were not statistically significant, likely due to smaller sample size, general trends towards dose-effect associations between the duration of breastfeeding and risk of earlier thelarche remained across racial/ethnic groups. The associations were stronger in African Americans than other race/ethnicity groups (not breastfed vs. breastfed > 6 months: HR:1.92; 95% CI: 1.01–3.66; breastfed < 6 months vs. > 6 months: HR: 1.63; 95% CI: 0.96–2.77). Similar trends between breastfeeding and onset was also seen in pubarche models among all racial/ethnic groups except non-Hispanic Whites.

**Table 5:** Association between breastfeeding duration and pubertal onset, stratified by maternal race/ethnicity: KPNC puberty study (*N* = 3331)

Race/Ethnicity	n	Breastfed ≥ 6 months	Breastfed < 6 months		Not breastfed	
		HR	HR	95% CI	HR	95% CI
Breast development^a^						
Non-Hispanic White	1387	Ref	1.15	0.95–1.40	1.19	0.92–1.56
African American	243	Ref	1.63	0.96–2.77	1.92	1.01–3.66
Asian/Pacific Islander	780	Ref	1.00	0.76–1.31	1.32	0.92–1.89
Hispanic	860	Ref	0.99	0.75–1.30	1.21	0.86–1.71
Combined	3270	Ref	1.09	0.97–1.22	1.25	1.07–1.46
Pubic hair development^a^						
Non-Hispanic White	1311	Ref	1.30	1.06–1.60	1.17	0.88–1.56
African American	227	Ref	0.99	0.48–2.02	1.14	0.51–2.52
Asian/Pacific Islander	740	Ref	1.24	0.94–1.64	1.33	0.93–1.91
Hispanic	809	Ref	0.88	0.67–1.16	1.30	0.93–1.82
Combined	3087	Ref	1.14	1.00–1.30	1.24	1.05–1.46

*CI* Confidence interval, *HR* Hazard ratio, *KPNC* Kaiser Permanente Northern California

^a^Adjusted for maternal age at delivery, education, and parity

## Discussion

In this multiethnic cohort of U.S. mother-daughter pairs, we found that breastfeeding is associated with timing of pubertal onset. Girls who were not breastfed are at increased risk of earlier breast and pubic hair onset compared with girls who breastfed for ≥6 months. Adjustment of the models with childhood BMI partially attenuated the associations, but they remained significant. Lastly, we observed that the association between breastfeeding and timing of thelarche may vary by race/ethnicity. There are several potential mechanisms explaining our observations.

The WHO guidelines on breastfeeding recommend that infants be exclusively breastfed for the first 6 months of life in order to ensure optimal health benefits for both mother and baby [25]. One of the known benefit of breastfeeding is lower risk of childhood obesity, which in turn is known to be associated with pubertal timing [1, 12]. These associations may be explained by the regulatory role of insulin in fat cell formation. Lucas et al. found that 6-day-old term infants that are formula-fed have significantly higher levels of blood insulin compared with breastfed infants. Insulin plays an important role in fat deposition by inhibiting lipolysis, the biological mechanism used to break down fats and other lipids. As a result, breastfed infants were found to have less body fat compared with formula-fed infants [33]. Additionally, breast milk has two regulatory hormones, leptin and ghrelin, which are involved in food intake and body weight regulation [34]. Breastfed infants also have more control over their energy intake compared with formula-fed infants and may be less likely to overeat [35]. Thus, breastfeeding provides a balance of nutrients and bioactive substances necessary to maintain healthy levels of fat. Although we do not have direct measures of adiposity or body composition, our results demonstrating the role of child prepubertal BMI suggest that obesity or higher fat may partially influence the association between breastfeeding and earlier pubertal onset. However, the significant association even after adjustment for pre-pubertal BMI suggest that there may also be other pathways explaining the associations between breastfeeding and pubertal timing.

Psychosocial factors may also explain the observed associations. The evolutionary-developmental theory of reproductive development postulates that early childhood experiences, such as infant-parent attachment, regulate reproductive development later in life [36]. One study found that infants with insecure infant-parent attachment had earlier pubertal onset compared with infants with secure attachment [36]. Infant-parent attachment security has been shown to have a positive association with breastfeeding duration and could mediate the relationship between breastfeeding and pubertal onset [37]. It is also possible that this relationship was confounded by maternal factors that are inversely associated with breastfeeding. For example, mothers with maternal depression and/or partner/marital stress may be less likely to initiate breastfeeding [38, 39]. Likewise, daughters raised in households affected by maternal depression or familial stress may be more likely to experience earlier pubertal timing [40]. Thus, not breastfeeding may be a proxy for maternal stress and depression, leading to earlier onset of daughters’ puberty. In this case, psychosocial interventions focusing on improving maternal mental health and wellness could potentially mitigate adverse health outcomes, such as earlier pubertal onset, in offspring later in life.

Our findings are consistent with the two existing studies that have examined breastfeeding and pubertal onset. We have recently conducted a study using a U.S.-based multiethnic cohort of 1237 girls and reported that girls who were predominantly breastfed were less likely to experience earlier thelarche compared with girls who were formula-fed (HR: 0.74; 95%CI: 0.59–0.94) [13]. Similarly, a study using a cohort of 219 Korean boys and girls found that being breastfed for at least 6 months compared with not being breastfed was inversely associated with earlier pubertal onset (odds ratio [OR]: 0.27; 95% CI: 0.11–0.66) [14]. Comparable results have been found in relation to menarche. Al-Sahab et al. found that an increase in 1 month of exclusive breastfeeding was associated with later menarche (HR: 0.94; 95% CI: 0.90–0.98) [17]. Similarly, another study showed that formula-fed girls were more than twice as likely to have experienced earlier menarche compared with breastfed girls [19]. Our results extend the existing knowledge by examining racial/ethnic differences. Although the test for interaction was not statistically significant, our data suggest that the strengths of the associations may vary by race/ethnicity. In our data, we observed particularly strong associations amongst African American girls. This may be partially due to the presence of greater variability in breastfeeding practice and duration amongst African Americans, as observed in Kale et al. [13], and warrants further investigation using a larger cohort given the known racial/ethnic disparities in the timing of pubertal onset. If racial/ethnic differences in these associations truly exist, such knowledge would help the design of future interventions involving breastfeeding and psychosocial support to reduce the risk of early puberty, thereby reducing ever-widening racial/ethnic health disparities.

There are several limitations to this study to be considered. First, reliance on data from the EHR and other clinical sources meant that detailed data were not available on potentially relevant factors. For example, although we were able to identify clear groups of mothers according to breastfeeding practices, our measure was relatively crude, and we were unable to provide more precise estimates of breastfeeding duration or exclusivity in our cohort. However, because of the availability of EHR data, including prospectively-collected pubertal stage data assessed objectively by pediatricians and clinically measured BMI, we were able to examine the associations of breastfeeding and pubertal onset in a large number of girls. Second, we did not have information on potential confounders or mediating factors such as girls’ body composition, maternal psychosocial factors such as stress or depression, parenting styles and breast milk composition. Lastly, we were only able to examine these associations amongst girls due to funding constraints. Early-life risk factors for pubertal onset are vastly understudied in boys. These are exciting areas for future research, and results from future studies may shed more light on the racial/ethnic differences in the timing of pubertal onset. Despite these limitations, our study extended the knowledge in the area of risk factors of early puberty by using diverse and large cohort of mother-daughter pairs, enabling us to explore racial/ethnic differences, which had never been done in previous studies.

## Conclusions

Breastfeeding may be an important independent modifiable factor in delaying breast and pubic hair onset in girls who are at higher risk of earlier pubertal onset. Potential race/ethnic variations in these associations as well as roles of maternal psychosocial factors need to be further explored in future studies. Results from such studies will inform design of future targeted interventions around breastfeeding and psychosocial support, which may confer additional health benefits to delay onset of puberty in high risk girls to promote subsequent beneficial psychosocial and health effects throughout the life course.

## Data Availability

The datasets generated and/or analyzed during the current study are not publicly available due to our institutional policy. Individuals who are interested in accessing the data may contact the corresponding author regarding [or to discuss or set up] a data use agreement.

## Abbreviations

BMI: Body mass index
CDC: Centers for Disease Control and Prevention
CI: Confidence interval
EHR: Electronic health record
HR: Hazard ratio
KPNC: Kaiser Permanente Northern California
OR: Odds ratio
SAS: Statistical Analysis System
TR: Time ratio
WHO: World Health Organization
