# Correction to: Breastfeeding and timing of pubertal onset in girls: a multiethnic population-based prospective cohort study

**DOI:** 10.1186/s12887-019-1671-8

**Published:** 2019-09-05

**Authors:** Sara Aghaee, Julianna Deardorff, Louise C. Greenspan, Charles P. Quesenberry, Lawrence H. Kushi, Ai Kubo

**Affiliations:** 1 Kaiser Permanente Division of Research, 2000 Broadway, Oakland, CA 94612 USA; 2 Division of Maternal and Child Health, University of California, Berkeley, School of Public Health, 2121 Berkeley Way #5302, Berkeley, CA 94720 USA; 3 Kaiser Permanente San Francisco Medical Center, 2425 Geary Boulevard, San Francisco, CA 94115 USA

**Correction to: BMC Pediatrics**

**https://doi.org/10.1186/s12887-019-1661-x**

Following publication of the original article [1], the authors reported that Table 4 was incorrectly presented. The revised and corrected version is shown below.

**Table 4:** Associations between breastfeeding duration and pubertal development, adjusted for prepubertal BMI: KPNC puberty study

Breastfeeding duration	Breast^a^			Pubic hair^a^		
	N	HR	95% CI	N	HR	95% CI
Not breastfed	497	1.18	1.01-1.38	470	1.13	0.96-1.34
< 6 months	1096	1.07	0.95-1.21	1023	1.12	0.98-1.28
≥ 6 months	1667	Ref		1587	Ref	

*CI* Confidence interval, *HR* Hazard ratio, *KPNC* Kaiser Permanente Northern California

^a^Also adjusted for maternal race/ethnicity, maternal age at delivery, education, and parity

The original article has been corrected.

## References

[1] Aghaee S, Deardorff J, Greenspan LC, Quesenberry CP, Kushi LH, Ai K (2019). Breastfeeding and timing of pubertal onset in girls: a multiethnic population-based prospective cohort study. BMC Pediatrics.

